# Prediction of the Bubble Growth Behavior by Means of the Time-, Temperature-, Pressure- and Blowing Agent Concentration-Dependent Transient Elongational Viscosity Function of Polymers

**DOI:** 10.3390/polym16091213

**Published:** 2024-04-26

**Authors:** Tobias Schaible, Christian Bonten

**Affiliations:** Institut für Kunststofftechnik, University of Stuttgart, Pfaffenwaldring 32, 70569 Stuttgart, Germany; christian.bonten@ikt.uni-stuttgart.de

**Keywords:** foaming, bubble growth simulation, transient uniaxial and equibiaxial elongational viscosity, elongational viscosity master curve, molecular stress function (MSF) model

## Abstract

Bubble growth processes are highly complex processes, which are not only dependent on the foaming process parameters (temperature, pressure and blowing agent concentration) but also on the type and structure of the polymer used. Since the elongational viscosity at the bubble wall during bubble growth also depends on these influencing factors, the so-called transient elongational viscosity plays a key role in describing the gas bubble growth behavior in polymer melts. The model-based description of the transient elongational viscosity function is difficult due to its dependence on time, Hencky strain and strain rate. Therefore, representative viscosities or shear viscosity models are usually used in the literature to predict the bubble growth behavior. In this work, the transient equibiaxial elongational viscosity function at the bubble wall during bubble growth is described holistically for the first time. This is achieved by extending the so-called molecular stress function (MSF) model by superposition principles (temperature, pressure and blowing agent concentration) and by using the elongational deformation behavior (Hencky strain and strain rate) at the bubble wall during the initial, and thus viscosity-driven, bubble growth process. Therefore, transient uniaxial elongational viscosity measurements are performed and the non-linear MSF model parameters of the two investigated polymers PS (linear polymer chains) and PLA (long-chain branched polymer chains) are determined. By applying the superposition principles and by changing the strain mode parameter to the equibiaxial case in the MSF model, the transient equibiaxial viscosity master curve is obtained and used to describe the bubble growth process. The results show that the extended MSF model can fully predict the transient equibiaxial elongational viscosity function at the bubble wall during bubble growth processes. The bubble growth behavior over time can then be realistically described using the defined transient equibiaxial elongational viscosity function at the bubble wall. This is not possible, for example, with a representative viscosity and therefore clearly demonstrates the influence and importance of knowing the transient deformation behavior that prevails at the bubble wall during bubble growth processes.

## 1. Introduction

Polymer foams are produced by adding blowing agents to the polymer melt and are therefore specifically manufactured two-phase systems (polymer and gas phase) [1]. In foam extrusion or foam injection molding, the physical blowing agent (gas formation through phase transition, which is relevant to this work) is injected directly into the polymer melt under high pressure, thus initially forming a two-phase system. The blowing agent is then dissolved in the polymer melt through sorption and diffusion processes, resulting in a single-phase solution [2]. 

Nucleation of the gas cell is initiated by the pressure drop caused in the mold or die, as the solubility of the blowing agent within the polymer melt is abruptly reduced due to pressure decrease [3]. The nucleated and growth-capable cell nuclei then begin to form bubbles. The bubbles formed continue to grow by diffusion processes until the bubble growth process is stopped due to a reduction in temperature caused by the cooling of the polymer melt in the process, resulting in an increase in the viscosity of the polymer melt [4]. Over the time of bubble growth, the driving force in the bubble must be greater than the clamping force at the bubble wall resulting from the elongational viscosity, and thus to the resistance to deformation of the blowing agent-loaded polymer melt [5,6,7].

**Figure 1:**
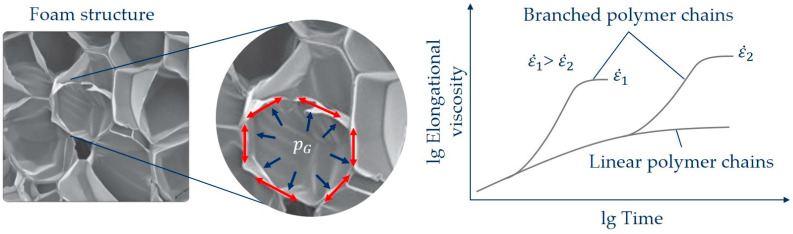
The role of the transient elongational viscosity at the bubble wall during bubble growth, according to [8].

If an exemplary bubble is observed during bubble growth, as shown schematically in Figure 1 (according to [8]), it becomes clear that a so-called transient equibiaxial deformation prevails at the bubble wall, and thus tensile or compressive forces occur in the bubble wall (red arrows in Figure 1), caused by the gas pressure in the bubble (blue arrows). With an idealized spherical assumption of the bubble, equibiaxial deformation is assumed. The elongational viscosity under a transient equibiaxial deformation thus plays a major role in bubble formation and growth over time [9].

Bubble growth processes mainly depend on the elongational deformation behavior of the polymer melt due to the structure of the polymer chains, as can be seen in Figure 1 (right) for two exemplary strain rates $\dot{\varepsilon }$. Long-chain branched polymers, meaning polymers with a branched polymer chain structure, exhibit a high increase in the transient elongational viscosity over time with increasing Hencky strain compared to unbranched polymers at the same strain rate. This so-called strain-hardening deformation behavior is caused by the fact that the long-chain branched polymer chains are less able to slide against each other and untangle under deformation [1,10]. During processing and foaming, the transient elongational viscosity also depends on the process conditions, and thus on the changes in temperature, pressure and blowing agent concentration over time [11].

In the simplest case, the so-called single-cell model [12,13,14,15,16,17], a model for describing the growth of an isolated, stable and growth-capable bubble within an influencing cell, can be used for the mathematical description of bubble growth over time. The single-cell model has also given rise to a number of model extensions, for example by Amon and Denson [12] (consideration of multiple and independent cells) or by Shafi et al. [13,15,17] (coupling of nucleation and bubble growth theory). However, the single-cell model is sufficient for investigating the influence of viscosity on bubble growth behavior [18].

**Figure 2:**
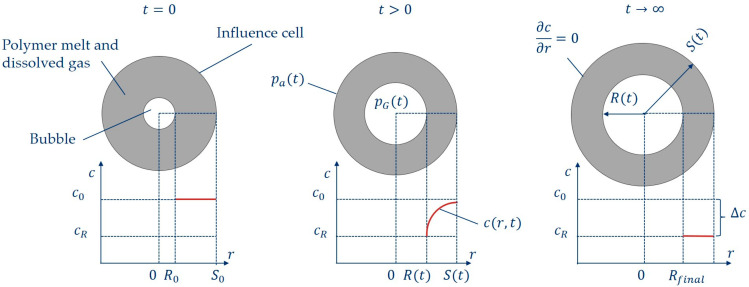
Schematic presentation of the single-cell model at three different times.

For the model concept, as shown in Figure 2 (according to [12,13,16]), a spherical gas bubble with the initial radius $R_{0}$ is present in a spherical influence cell volume with radius $S_{0}$ at time t=0 with the average dissolved blowing agent concentration $c_{0}$ within the influence cell. The ambient pressure $p_{a}(t)$ acts on the influnece cell. At time t>0 the bubble radius R(t) and the influence cell radius S(t) increase due to the pressure difference between the gas pressure within the bubble $p_{G}(t)$ and the ambient pressure $p_{a}(t)$ as well as by the diffusion mass flow of the blowing agent-loaded polymer melt into the gas phase in the bubble. This results in a concentration gradient between the bubble radius and the influence cell radius depending on the distance r and time t. Furthermore, the simplification is made that no diffusion or gas exchange to neighboring influence cells is permitted outside the influence cell (∂c/∂r=0). At time t→∞, the final bubble radius $R_{final}$ is reached and the available blowing agent concentration ∆c has diffused into the gas phase within the bubble.

The description of the diffusion-controlled and viscosity-driven bubble growth process in Figure 2 requires the solution of a complex system of differential equations consisting of the combined momentum and continuity equation (Equation (1)), the gas mass balance at the bubble wall (Equation (2)) and Fick’s second law (Equation (3)).

Based on the pressure balance for the influence cell, the gas pressure within the bubble $p_{G}(t)$ can be described as the sum of the ambient pressure $p_{a}(t)$ on the influence cell, the pressure component from the surface tension $\sigma_{O}$ at the interface of the bubble wall and the dynamic pressure within the blowing agent-loaded polymer melt for each time during bubble growth [19].

(1) $$p_{G}(t)=p_{a}(t)+\frac{2\cdot \sigma_{O}}{R(t)}+4\cdot \frac{\partial R}{\partial t}\cdot R^{2}\left(t\right)\cdot \int_{z\left(R\left(t\right)\right)}^{z\left(S\left(t\right)\right)}\eta \left(z\left(t\right)\right)\cdot dz$$

In order to describe the dynamic pressure, the viscosity integral $\eta \left(z\left(t\right)\right)$ is set up with the spatial coordinate $z=1/r^{3}$ as a function of the radius r between $R\left(t\right)$ and $S\left(t\right)$. This describes the change in viscosity within the influence cell in terms of the spatial coordinate (change in blowing agent concentration) and time (change in time-dependent deformation).

The mass balance requires that the change in mass of gas in the bubble is equal to the diffusion mass flow through the bubble surface. Thus, the gas pressure is related to the concentration gradient ${\left(\partial c/\partial r\right)}_{r=R}$ at the bubble wall [19].

(2) $$\frac{d}{dt}\left(\frac{4\cdot \pi \cdot {R(t)}^{3}}{3}\cdot \frac{p_{G}(t)\cdot M}{R_{G}\cdot T}\right)=4\cdot \pi \cdot {R(t)}^{2}\cdot D_{0}\cdot \rho \cdot {\left(\frac{\partial c}{\partial r}\right)}_{r=R}$$

The density of the polymer melt is described by ρ and its diffusion coefficient by $D_{0}$, the temperature by T, the molar mass of the blowing agent by M and the universal gas constant by $R_{G}$. The concentration gradient at the bubble wall is described by means of Fick’s second law in Equation (3). In this way, the spatial coordinate-dependent and time-dependent concentration profile of the blowing agent within the influence cell is described [13].

(3) $$\frac{\partial c}{\partial t}+\frac{\partial R}{\partial t}\cdot {\left(\frac{R\left(t\right)}{r}\right)}^{2}\cdot \frac{\partial c}{\partial r}=\frac{D_{0}}{r^{2}}\cdot \frac{\partial }{\partial r}\cdot \left(r^{2}\cdot \frac{\partial c}{\partial r}\right)\;\;\;;\;r\geq R$$

In the literature, simplifying assumptions are often made to describe the viscosity or viscosity function η(z(t)) in Equation (1), since the description of the transient equibiaxial elongational viscosity function at the bubble wall during bubble growth is a big challenge. In addition, the dependencies described above, such as the constitution of the polymer chain (linear or branched polymer chains), the changing Hencky strain as well as the strain rate at the bubble wall during bubble growth over time and the process conditions (temperature, pressure and blowing agent concentration) must be considered in a transient elongational viscosity model.

Zhang et al. [20], Ataei et al. [21] and Leung et al. [22] considered the changing viscosity using a zero-shear viscosity approach as a function of temperature, pressure and blowing agent concentration according to Lee et al. [23]. Thus, a changing and representative viscosity, based on the zero-shear viscosity, was determined during bubble growth. A similar approach based on the changing zero-shear viscosity was also used by Ferasat et al. [24]. Furthermore, Leung et al. [22], Han et al. [25] and Kim et al. [26] were able to show by simulation that the relaxation times of the polymer melt significantly influence the effect of viscosity on bubble growth. The influence of viscosity is particularly pronounced at low relaxation times. Ramesh et al. [27] used and compared models assuming an effective zero-shear viscosity, a power law approach and a viscoelastic model to describe the viscosity. They found that the viscoelastic model best represented the observed bubble growth in an autoclave process. In addition, Shimoda et al. [28] described a shear viscosity model based on a power law approach that showed a dependence on temperature, shear rate and blowing agent concentration. Similarly, Breuer et al. [29] attempted to predict the bubble growth in foam extrusion. They used the Carreau approach in combination with the WLF–Chow model [30] taking the influence of blowing agent, pressure and temperature on shear viscosity into account.

Until today, no description of the transient equibiaxial elongational viscosity function during the bubble growth process as a function of Hencky strain, strain rate, pressure, temperature and blowing agent concentration has been reported in the literature. This is also confirmed by Wang (2009) [9], Raps et al. (2017) [31], Ataei et al. (2019) [21] and Yao et al. (2021) [32].

Therefore, in this work a rheological model based on the so-called molecular stress function (MSF) model [33,34,35] and its extension by superposition principles is presented. With this model and with the knowledge of the prevailing deformation behavior at the bubble wall during bubble growth, the transient equibiaxial elongational viscosity function for bubble growth over time can be described holistically for the first time. Finally, it is possible to describe the initial, and thus viscosity-driven, bubble growth behavior, which starts immediately after nucleation with a viscosity-driven and diffusion-controlled bubble growth model.

The methodology and models for the application of superposition (temperature, pressure and blowing agent concentration) on viscosity [11] and the determination of the transient expansion and thus deformation behavior (Hencky strain and strain rate) during bubble growth at the bubble wall [36] have already been published by the authors and are taken up in this work.

## 2. Materials and Methods

### 2.1. Materials

The amorphous polystyrene PS 168N (linear polymer chains) from Ineos Styrolution Group GmbH, Frankfurt am Main, Germany, and the semi-crystalline polylactide PLA IngeoTM biopolymer 2003D from NatureWorks LLC, Minnetonka, MN, USA, were used in this work for analysis. PS is a widely used polymer for foam applications in various industrial sectors, while PLA is becoming more and more important as a bio-based and bio-degradable polymer for foam applications. Due to the low viscosity and poor melt strength of PLA, it must be modified for foam applications [8]. After modification, PLA shows an increased viscosity and strain-hardening under deformation. The modification (according to [8], developed at the IKT University of Stuttgart) therefore changes the constitution of the polymer chains from a linear (unbranched) to a branched or long-chain branched constitution [11,37].

### 2.2. Rheological Characterization and Superposition

For rotational rheometric characterization of PS and PLA, the Discovery HR-2 rotational rheometer from TA Instruments, New Castle, DE, USA, was used in a plate–plate setup. Initially, the linear viscoelastic deformation range was determined using the so-called deformation sweep. The deformation was set to 5% for all measurements within the linear viscoelastic limit. To analyze the complex viscosity as a function of shear rate and temperature, measurements were carried out using the so-called frequency sweep in the shear rate range between 628 rad/s and a minimum of 0.001 rad/s. The minimum measurable shear rate was adjusted depending on the temperature (PS: 180 °C, 200 °C, 220 °C, 240 °C and PLA: 180 °C, 200 °C, 220 °C) and the type of polymer, so that no thermal degradation occurred over the measurement time and the zero-shear viscosity was recorded.

The transient uniaxial elongational viscosity was characterized with the SER (Sentmanat extensional rheometer) measurement setup according to [38] from TA Instruments, New Castle, DE, USA, using the rotational rheometer. Test specimens (length 18 mm, thickness 0.7 mm and width 10 mm) were manufactured by compression molding. The same temperatures as for the frequency sweep were applied over the Hencky strain range from 0 to 3.8 and at the three exemplary strain rates 12 s^−1^, 2 s^−1^ and 0.05 s^−1^ for examinations. All measurements were performed three times at each measurement setting. The mean values from the three measurements are shown in Section 4.

The superposition principles were determined from in-line shear viscosity measurements in the physical foam injection molding process as a function of the shear rate, temperature, pressure and blowing agent concentration. The experimental setup and the superposition models used to describe the effect of temperature, pressure and blowing agent concentration on viscosity have been previously published by the authors in [11] and are taken up in this work.

### 2.3. In-Line Analysis of Bubble Growth and Transient Deformation Behavior over Time

The experimental setup used for the visual in-line observation of the initial, highly dynamic and transient deformation and expansion behavior of the blowing agent in the blowing agent-loaded polymer melt was published by the authors in [36] and is taken up in this work. For this purpose, the water box of an underwater pelletizing process was modified in such a way that the die outlet, and thus the expansion and deformation behavior of the bubble growth over time, can be observed in-line during the foam extrusion process. N_2_ was added as a blowing agent in the extrusion process, and therefore the expansion and deformation behavior at the die outlet were analyzed at a back pressure of approx. 1 bar water pressure in the water box and under almost isothermal conditions during the cut of the pelletizer (cutting time approx. 6.7 ms).

Based on the expansion behavior shown in [36], and thus the total gas volume present in the blowing agent-loaded polymer melt over time of bubble growth, the mean initial bubble growth behavior as a function of the process conditions and the polymer used were determined by means of the mean number of bubbles per foamed pellet. For this purpose, the assumptions were made that all nucleated bubbles grow spherically and that coalescence effects and the decay of bubbles can be neglected on average.

The FF20CT microfocus system from YXLON International GmbH, Hamburg, Germany, in combination with the Varex 2530HE detector (resolution 2146 × 1762 pixels) was used for the CT analyses of the foamed pellets after underwater pelletizing to analyze the average number of bubbles in the foamed pellet.

## 3. Transient Elongational Viscosity Model and Bubble Growth Simulation

### 3.1. Molecular Stress Function Model Extension by Superposition

The molecular stress function (MSF) model in Equation (4), which is well described in the literature [35,39,40,41,42,43,44,45,46] represents a generalized form and further development of the model according to Doi and Edwards (DE) and is used to predict the transient elongational viscosity. The linear viscoelastic memory function $m\left(t-t^{\prime }\right)$ and the time-dependent non-linear viscoelastic deformation measure ${\underline{\underline{S}}}_{MSF}(t,t^{\prime })$ are used to describe the current stress state $\underline{\underline{\sigma }}\left(t\right)$ of a volume element at a certain time, taking the deformation history in the time period from time $t^{\prime }=-\infty$ (undeformed state) to the currently deformed state $t^{\prime }=t$ (observation time) into account. Finally, the transient elongational viscosity $\mu \left(t,\dot{\varepsilon }\right)=\sigma (t,\dot{\varepsilon })/\dot{\varepsilon }$ can be calculated using the current stress state and the strain rate $\dot{\varepsilon }$.

(4) $$\underline{\underline{\sigma }}\left(t,\dot{\varepsilon }\right)=\int_{t^{\prime }=-\infty }^{t^{\prime }=t}m(t-t^{\prime })\cdot {\underline{\underline{S}}}_{MSF}(t,t^{\prime })\cdot dt^{\prime }$$

The basic assumption of the MSF model, as with all so-called tube models, is that the polymer chains are limited in their ability to move due to the surrounding entanglements of the neighboring polymer chains and that they cannot penetrate each other [1,39]. It is therefore assumed that the movement of an isolated polymer chain is limited within its resulting tube, meaning the free volume.

The linear viscoelastic memory function $m\left(t-t^{\prime }\right)$ according to Equation (5) is obtained from rotational rheometric measurements in the linear viscoelastic deformation range and is described by the time derivative of the time-dependent linear viscoelastic shear relaxation modulus $G(t-t^{\prime })$ using the discrete relaxation time spectrum. The relaxation strength is presented by $g_{i}$ and the relaxation time by $\lambda_{i}$ [47,48,49].

(5) $$m\left(t-t^{\prime }\right)=\frac{dG(t-t^{\prime })}{dt^{\prime }}=\sum_{i=1}^{N}\left(\frac{g_{i}}{\lambda_{i}}\right)\cdot e^{-\frac{\left(t-t^{\prime }\right)}{\lambda_{i}}}$$

The time-dependent non-linear deformation measure of the MSF model ${\underline{\underline{S}}}_{MSF}(t,t^{\prime })$ is described by Equation (6) according to [34,39,41], using the quadratic molecular stress function $f^{2}$ and the deformation measure ${\underline{\underline{S}}}_{DE}^{IA}(t,t^{\prime })$ according to DE.

(6) $${\underline{\underline{S}}}_{MSF}(t,t^{\prime })=5\cdot f^{2}\cdot {\left\langle \left.\frac{\underline{u^{\prime }}\cdot \underline{u^{\prime }}}{{u^{\prime }}^{2}}\right\rangle \right.}_{0}=f^{2}\cdot {\underline{\underline{S}}}_{DE}^{IA}(t,t^{\prime })$$

Therefore, IA stands for the independent alignment (IA) and means that the segments of a polymer chain, and thus its tangential vector, can be deformed independently of those of other segments of a polymer chain [39]. The quadratic molecular stress function $f^{2}$ in the MSF model represents a correction of the damping function according to the DE model [33], since in the MSF model the tube diameter and the tube length are variable due to the occurring deformation. The time-dependent rate of change of the quadratic molecular stress function of the MSF model can be described using Equation (7) for polydisperse (polymers with a molar mass distribution), linear (unbranched) and branched polymers. A detailed description can be found in the literature [34,39,40,41].

(7) $$\frac{\partial f^{2}}{\partial t}=\dot{\varepsilon }\frac{\beta \cdot f^{2}}{1+\frac{\beta -1}{f^{4}}}\left(S_{11}+m_{E}\cdot S_{22}-(1+m_{E})\cdot S_{33}\;\;\;\;\;\;\;\;\;\;\;\;\;-\frac{f^{2}-1}{f_{max}^{2}-1}\sqrt{S_{11}+m_{E}^{2}\cdot S_{22}+{\left(1+m_{E}\right)}^{2}\cdot S_{33}}\right)$$

The non-linear parameter β is a measure that represents the average number of branched chain segments (side chains) of the same so-called backbone (main chain) within the tube [46]. Thereby, 1/β describes the proportion of stretched chain segments [50]. Experimental investigations have shown that β describes the slope of the transient elongational viscosity function from the beginning of the strain-hardening deformation behavior [34,46]. In the case of polydisperse linear (unbranched) polymers, β = 1, which results in the so-called LMSF model (linear MSF model) [41]. However, the model according to DE results for β = 1 and $f_{max}^{2}$ = 1 [34]. Depending on the degree of branching, values up to β = 4 have been determined in the literature for polydisperse and long-chain branched polymers [34,51]. $f_{max}^{2}$ corresponds to the theoretical stationary value of the elongational viscosity and thus describes the maximum possible and stored energy in the polymer chain system under a non-linear viscoelastic elongational deformation [44,51]. Thereby, $\dot{\varepsilon }$ corresponds to the strain rate in the main direction along the main chain and $S_{11}$ to $S_{33}$ are the corresponding components of the orientation tensor $\underline{\underline{S}}$ in the direction parallel $S_{11}$, transverse $S_{22}$ and perpendicular $S_{33}$ to the stretching direction [34].

In the case of linear (unbranched) polymers, the assumptions are valid for describing the deformation of the main chain along the so-called primitive path. To describe branched or long-chain branched polymers, it is assumed that the main chain in the tube is stretched during deformation and the side chains in the tube are compressed [46]. Since this mechanism is independent of the type of deformation (uniaxial or equibiaxial) the MSF model can be calibrated using transient uniaxial elongational viscosity measurements. The calibration is used to determine the non-linear MSF model parameters. The type of deformation (uniaxial: $m_{E}$ = −0.5 and equibiaxial: $m_{E}$ = 1) is considered by changing the deformation mode parameter $m_{E}$.

Once the transient uniaxial calibration of the MSF model has been performed, the MSF model can be extended with the superposition principles $a_{T}$ (time–temperature shift), $a_{p}$ (time–pressure shift) and $a_{c}$ (time–blowing agent concentration shift).

The superposition principles described under a shear deformation [11] (previously published by the authors) are coupled with the transient uniaxial elongational viscosity function $\mu_{u}\left(t,\dot{\varepsilon },T,p,c\right)$ as a function of temperature T, pressure p and blowing agent concentration c as follows (see Equation (8); index 0 describes the reference condition).

(8) $$\mu_{u}\left(t,\dot{\varepsilon },T,p,c\right)=a_{T}\cdot a_{p}\cdot a_{c}\cdot \mu_{u}\left(t\cdot a_{T}\cdot a_{p}\cdot a_{c},\;\dot{\varepsilon },\;T_{0},p_{0},c_{0}\right)$$

Using Equation (8), a master curve of the transient uniaxial SER measurement data can be generated. In addition, the superposition must also be applied to the strain rate according to Equation (9) [52].

(9) $$\dot{\varepsilon }\left(T,p,c\right)=\frac{\dot{\varepsilon }\left(T_{0},p_{0},c_{0}\right)}{a_{T}\cdot a_{p}\cdot a_{c}}$$

The master curve of the transient uniaxial SER measurement data can then be predicted by the transient uniaxial calibrated and extended MSF model for all strain rates. If this is given, the transient uniaxial calibrated MSF model can be transferred to the case of an equibiaxial deformation and its master curve $\mu_{b}\left(t,\dot{\varepsilon },T,p,c\right)$ prediction according to Equation (10) by changing $m_{E}$ to the equibiaxial case.

(10) $$\mu_{b}\left(t,\dot{\varepsilon },T,p,c\right)=a_{T}\cdot a_{p}\cdot a_{c}\cdot \mu_{b}\left(t\cdot a_{T}\cdot a_{p}\cdot a_{c},\;\dot{\varepsilon },T_{0},p_{0},c_{0}\right)$$

### 3.2. Bubble Growth Simulation Using the Single-Cell Model

Finally, using the calibrated and extended MSF model and taking the determined deformation behavior (Hencky strain $\varepsilon \left(t\right)$ and strain rate $\dot{\varepsilon }\left(t\right)$, published by the authors in [36]) during initial, and thus viscosity-driven, bubble growth into account, the transient equibiaxial elongational viscosity function at the bubble wall can be accurately described and predicted. The transient equibiaxial elongational viscosity function at the bubble wall during the initial bubble growth over time is then consequently used in the bubble growth model. Thus, the initial viscosity-driven bubble growth behavior can be investigated and predicted for the first time as a function of the transient equibiaxial elongational viscosity function at the bubble wall over time.

In order to solve the system of differential equations (Equation (1) to Equation (3)) with the software MatLab Version R2020b, The MathWorks Inc., Natick, MA, USA, the following assumptions were made: The ideal gas law applies inside the bubble and the gas pressure $p_{G}(t)$ acts on the bubble wall. The gas pressure is related to the spatial coordinate- and time-dependent blowing agent concentration at the bubble wall via Henry’s law. It is also assumed that the polymer melt is incompressible, inertia effects are negligible, the material properties (e.g., diffusion coefficient or surface tension) are independent of the blowing agent concentration, the temperature and the pressure, the blowing agent concentration is homogeneously dissolved in the influence cell and the initial growth process is isothermal [13,16,19].

A detailed explanation of the necessary governing equations and their initial and boundary conditions for spherical coordinates to describe bubble growth using the single-cell model can be found in the literature [13,16,27,53]. The necessary physical input values of bubble growth prediction in Table 1 were either measured or taken from the literature according to the process conditions of the foaming experiments in [36]. In addition, the initial value of the influence cell radius $S_{0}$ was chosen in such a way that the resulting mean bubble radius from the experiments in [36] is represented due to the physical input values of the bubble growth model. The resulting bubble radius is in addition independent of the choice of the initial value of the bubble radius $R_{0}$. This procedure is also described in the literature [19].

## 4. Results and Discussion

### 4.1. Calibration of the Transient Uniaxial MSF Model for PS and PLA

The linear viscoelastic memory function $m\left(t-t^{\prime }\right)$ and the non-linear viscoelastic deformation measure ${\underline{\underline{S}}}_{MSF}(t,t^{\prime })$ must be determined to describe the measured transient uniaxial elongational viscosity with the MSF model. This requires a uniaxial calibration of the non-linear MSF model parameters β and $f_{max}^{2}$. The rheology software IRIS Version 2020 [60,61], version 2020, Amherst, MA, USA, was used for this purpose and to solve the MSF model equation (see Equation (4)).

**Figure 3:**
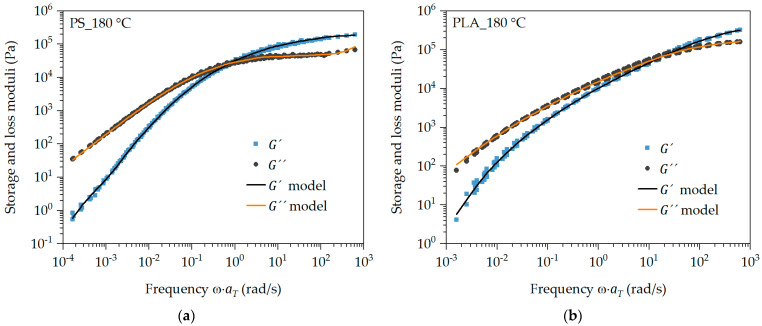
Storage and loss moduli measured and approximated by the discrete relaxation time spectrum of PS (**a**) and PLA (**b**) at reference temperature 180 °C.

The linear viscoelastic memory function $m\left(t-t^{\prime }\right)$ is determined from rotational rheometric measurements in the linear viscoelastic deformation range. For this purpose, a master curve of the storage and loss moduli is created over a wide temperature and shear rate range, whereby the discrete relaxation time spectrum is obtained. This is shown as an example for the master curve with the reference temperature of 180 °C for PS and PLA in Figure 3.

The non-linear MSF model parameters β and $f_{max}^{2}$ must be identified to describe the measured transient uniaxial elongational viscosity. β and $f_{max}^{2}$ must be chosen in such a way that the transient uniaxial elongational viscosity can be described and predicted with unchanged parameters over the widest possible temperature, Hencky strain and strain rate range. If this is the case, the principle of superposition automatically applies [62], resulting in a transient uniaxial calibration of the MSF model. This allows the transient equibiaxial elongational viscosity function to be predicted [41].

If β = $f_{max}^{2}$ = 1, then the model is simplified to that of DE. The linear MSF model (LMSF) results from β = 1 and $f_{max}^{2}$ > 1, whereas the MSF model is applied for β > 1 and $f_{max}^{2}$ > 1 (see Table 2). The difference is that, in the LMSF model, the stress prediction is corrected by the modified description of the damping function for unbranched and polydisperse polymer melts compared to the DE model. In contrast, the MSF model additionally considers the average number of branched chain segments of the same backbone in the tube by β > 1, allowing the transient elongational viscosity of polydisperse and branched polymer melts to be predicted.

**Figure 4:**
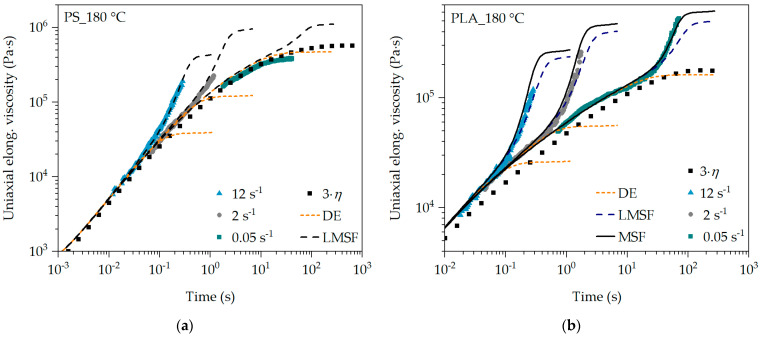
Analysis of the non-linear MSF model parameters for PS (**a**) and PLA (**b**) at 180 °C.

Since PS is a polymer with linear and therefore unbranched polymer chains, the DE and LMSF models are applied. While PLA has branched polymer chains after the modification, the application of the DE, LMSF and MSF models should confirm this and demonstrate the sensitivity of the MSF model. The prediction of the transient uniaxial elongational viscosity as a function of the non-linear MSF model parameters is shown as an example for PS and PLA at 180 °C in Figure 4.

**Figure 5:**
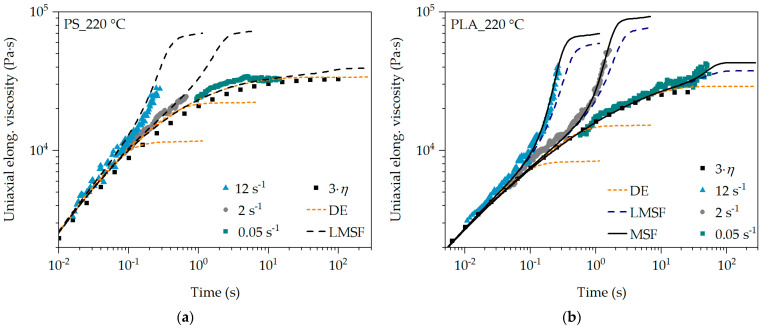
Analysis of the non-linear MSF model parameters for PS (**a**) and PLA (**b**) at 220 °C.

It is evident that the DE model represents the transient uniaxial elongational viscosity very well for PS in the range in which the Trouton ratio (3·*η*) is valid. With increasing time or Hencky strain, the transient uniaxial elongational viscosity curve is significantly underestimated with the DE model, as the prevailing stress is reduced too much due to insufficient representation of the damping function. Only for PS and very low strain rates of 0.05 s^−1^, the DE model provides an accurate prediction, as shown in Figure 4a and Figure 5a. The same can be observed for PLA. However, the uniaxial Trouton ratio for PLA deviates slightly from the SER measurements and the predictions at 180 °C. In Figure 5b, however, there is very good agreement at 220 °C for all strain rates in the range of low Hencky strains.

The LMSF model describes the elongational viscosity curve of PS at the exemplary strain rates of 2 s^−1^ and 12 s^−1^ in Figure 4a very well, whereas at 0.05 s^−1^ the elongational viscosity is overestimated at higher Hencky strains. A very good prediction of the SER measurements by the LMSF model for PS is obtained at 220 °C for all exemplary strain rates in Figure 5a. However, the transient uniaxial elongational viscosity curve of PLA is slightly underestimated with the LMSF model at 180 °C, especially at low strain rates (see 0.05 s^−1^ in Figure 4b). At 220 °C in Figure 5b, the LMSF model underestimates the elongational viscosity even at high strain rates of 12 s^−1^ and increasing Hencky strain. This can be attributed to the fact that the LMSF model does not consider the branched polymer chain architecture in PLA due to β = 1. Using the MSF model, on the other hand, the transient uniaxial elongational viscosity curve of PLA is precisely predicted at all strain rates and temperatures.

**Figure 6:**
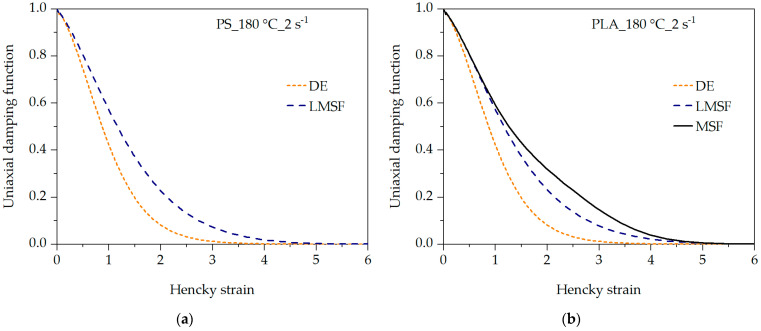
Analysis of the uniaxial damping function of the DE, LMSF and MSF models for PS (**a**) and PLA (**b**) at 180 °C and 2 s^−1^.

The differences in the prediction quality are based on the respective model assumptions (see Table 2). The DE model assumes no branching (β = 1) and a constant tube diameter ($f_{max}^{2}$ = 1), whereas, in the LMSF and MSF models, the tube diameter is reduced as a function of time ($f_{max}^{2}$ > 1) due to the deformation and thus the stretching of the polymer chains. In the case of the branched PLA, the branching of the polymer chains is considered by the MSF model with β > 1. The damping function corrects the stress prediction for non-linear deformation, meaning high and increasing deformation, by multiplicatively reducing the stress level according to the damping function [41,63]. Since the damping function of the DE model in Figure 6 is lower than that of the LMSF and MSF models, a higher stress reduction occurs, whereby the elongational viscosity is underestimated with an increasing Hencky strain.

Due to the fact that, with a well-defined parameter setting of β and $f_{max}^{2}$, the transient uniaxial elongational viscosity of PS and PLA can be described and predicted by the LMSF and MSF models over the entire temperature, strain rate and Hencky strain range (see Figure 4 and Figure 5), the superposition principle applies automatically [62]. Thus, the transient uniaxial calibration of the LMSF and MSF models for their application in transient equibiaxial elongational flows was obtained, whereby the transient uniaxial elongational viscosity can be described and predicted for all temperatures, Hencky strains and strain rates.

### 4.2. Master Curve of the Transient Uniaxial Elongational Viscosity

**Figure 7:**
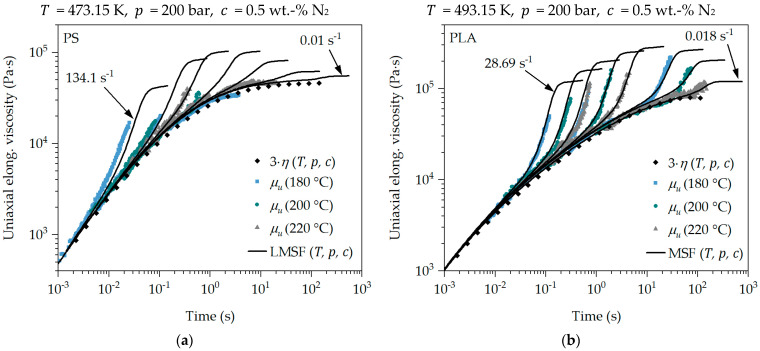
Master curve of the transient uniaxial elongational viscosity of PS (**a**) and PLA (**b**).

The combination of the transient uniaxial calibrated LMSF and MSF models with the superposition principles [11] (previously published by the authors) according to Equation (8) is used to describe the elongational deformation behavior of the blowing agent-loaded polymer melt. For this purpose, the superposition principles determined under a shear deformation are transferred to an elongational deformation. This is shown for the SER measurements at strain rates of 12 s^−1^, 2 s^−1^ and 0.05 s^−1^ for PS and PLA in Figure 7. By applying the relationship in Equation (8), the transient uniaxial elongational viscosity measurement data (SER measurements) in Figure 4 and Figure 5 can be shifted to any value of temperature, pressure and blowing agent concentration based on their reference conditions (SER measurement conditions: $T_{0}$ according to the SER measuring temperature, $p_{0}$ = 1 bar and $c_{0}$ = 0 wt.-% N_2_). Due to the transient representation of the elongational viscosity, the SER measurements are shifted under +45°. In Figure 7, the transient uniaxial master curve of the elongational viscosity $\mu_{u}\left(t,\dot{\varepsilon },T,p,c\right)$ results from the shifted SER measurement data as a function of time, strain rate, temperature, pressure and blowing agent concentration at the selected conditions regarding T, p and c. It is important to note that the superposition must also be applied to the strain rate according to Equation (9), which results in a significantly wider strain rate range compared to the measurable one.

If the calibrated LMSF and MSF models are now coupled with the superposition principles according to Equation (8), the transient uniaxial master curve of the elongational viscosity measurements can be precisely described and predicted at any strain rate. This proves the application of the superposition determined under a shear deformation and the transfer to an elongational deformation, as well as the coupling with the LMSF and MSF models. This is clearly shown in Figure 7 by the fact that the shifted transient uniaxial elongational viscosity measurements form a uniform master curve of the transient uniaxial elongational viscosity at the selected conditions with respect to T, p and c. In addition, the agreement with the shifted uniaxial Trouton ratio 3·$\eta \left(T,p,c\right)$ is within the applicable and previously discussed limits. Furthermore, the shifted transient uniaxial elongational viscosity measurements can be accurately described and predicted with the calibrated and coupled LMSF and MSF models at the selected conditions with respect to T, p and c for PS and PLA. This even applies to very high strain rates of approx. 134 s^−1^ in Figure 7a.

### 4.3. Prediction of the Transient Equibiaxial Elongational Viscosity Function at the Bubble Wall during Bubble Growth

**Figure 8:**
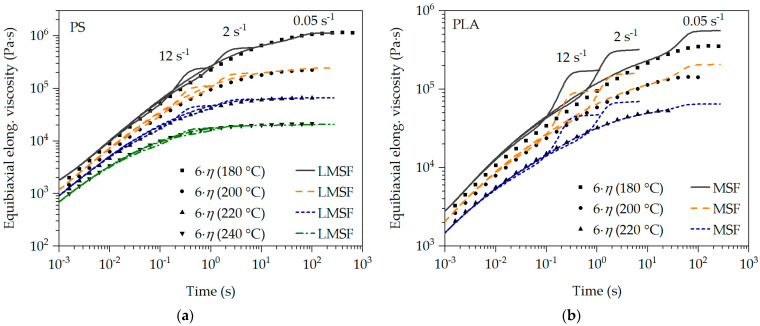
Prediction of transient equibiaxial elongational viscosity for PS (**a**) and PLA (**b**).

The described and demonstrated transient uniaxial calibration of the LMSF and MSF models can also be used to predict the transient equibiaxial elongational viscosity. For this purpose, the mode of deformation is set to $m_{E}$ = 1 [41]. The prediction of the transient equibiaxial elongational viscosity function for PS and PLA is shown in Figure 8. In addition, the equibiaxial Trouton ratio (6·η) as a function of time is shown as well.

The measurement of the transient equibiaxial elongational viscosity is highly demanding and complex, which is why no measurements could be carried out as part of this work. However, it can be seen in Figure 8 that the transient equibiaxial predictions (as with the uniaxial elongational viscosity predictions) correspond to the equibiaxial Trouton ratio at all strain rates and low Hencky strains and deviate from it as the Hencky strain increases. This is particularly evident with PLA in Figure 8b. For PS in Figure 8a, on the other hand, the equibiaxial Trouton ratio is exactly described by the predictions at low strain rates. In the case of PLA, it can also be seen that the strain-hardening deformation behavior is less pronounced with increasing temperature and decreasing strain rate. This was also observed in the measured transient uniaxial elongational viscosity curves. This confirms the plausibility of the predictions in Figure 8. This is additionally supported by the holistic transient uniaxial calibration of the LMSF and MSF models, whereby the transient equibiaxial elongational viscosity can be predicted without restrictions with the LMSF and MSF models for all considered temperatures and strain rates [41].

Following the proof of the relationships for the transient uniaxial elongational viscosity $\mu_{u}\left(t,\dot{\varepsilon },T,p,c\right)$ according to the model in Section 3.1, the transfer to the transient equibiaxial elongational viscosity $\mu_{b}\left(t,\dot{\varepsilon },T,p,c\right)$ as a function of time, strain rate, temperature, pressure and blowing agent concentration is now carried out. For this purpose, the relationship accordance to Equation (10) is applied. The data basis for this is provided by the LMSF and MSF model predictions at the respective reference temperatures $T_{0}$ (PS: 180 °C, 200 °C, 220 °C, 240 °C and PLA: 180 °C, 200 °C, 220 °C), the reference pressure $p_{0}$ = 1 bar and the reference blowing agent concentration $c_{0}$ = 0 wt.-% N_2_ in Figure 8.

**Figure 9:**
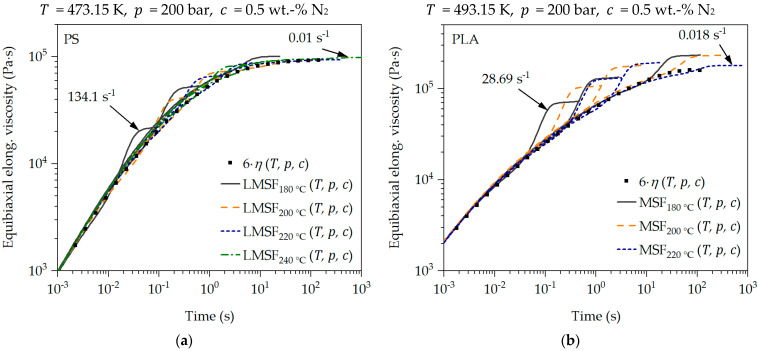
Master curve of the transient equibiaxial elongational viscosity of PS (**a**) and PLA (**b**).

The prediction of the transient equibiaxial elongational viscosity master curve $\mu_{b}\left(t,\dot{\varepsilon },T,p,c\right)$ at the chosen conditions with respect to T, p and c is shown in Figure 9. Even under a temperature-, pressure- and blowing agent concentration-dependent transient equibiaxial viscosity described by the LMSF and MSF model predictions $\mu_{b}\left(t,\dot{\varepsilon },T,p,c\right)$, the superposition principles are valid concerning the exemplarily selected conditions with respect to T, p and c in Figure 9. This is clearly shown by the superposition of all predictions in Figure 9 for the selected conditions, based on the reference conditions in Figure 8. Furthermore, the comparison of the transient equibiaxial LMSF and MSF model predictions with the corresponding equibiaxial Trouton ratio 6·$\eta \left(T,p,c\right)$ at the selected conditions with respect to T, p and c in Figure 9 shows a clear agreement within the valid limits of the Trouton ratio. Thus, the coupling of the LMSF and MSF models with the superposition principles is fully proven in Figure 7 for the transient uniaxial and in Figure 9 for the transient equibiaxial elongational viscosity function prediction.

**Figure 10:**
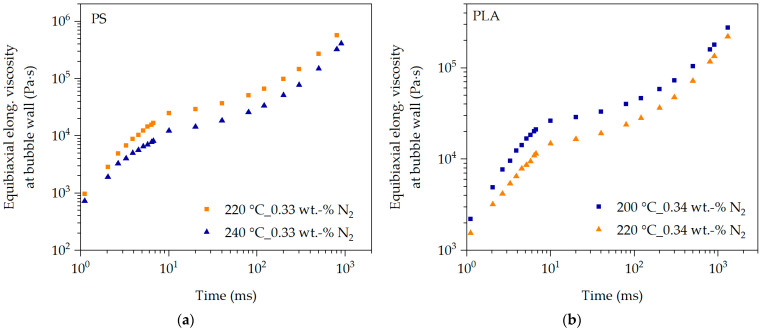
Transient equibiaxial elongational viscosity function at the bubble wall during bubble growth, described with the extended LMSF model for PS (**a**) and MSF model for PLA (**b**).

In Figure 10, the transient equibiaxial elongational viscosity function at the bubble wall over the time of bubble growth is described using the elongational deformation in the foam extrusion process, which was previously published by the authors in [36]. The Hencky strain and strain rate data at the bubble wall are used to describe the transient equibiaxial elongational viscosity during bubble growth using the calibrated and extended LMSF and MSF models according to Equation (10).

From the time (>6.7 ms in Figure 10) at which no more deformation data are available in [36], superposition is used to calculate the equibiaxial elongational viscosity function regarding temperature, pressure and blowing agent concentration during bubble growth. However, the influence of temperature is predominant, as the blowing agent-loaded polymer melt is cooled in the extrusion process after the die outlet. This causes the viscosity to increase significantly due to the influence of temperature reduction [11]. The mean caloric temperature within the foamed pellets was calculated using the model in [64].

Figure 10 clearly shows that the equibiaxial elongational viscosity function at the bubble wall increases significantly during bubble growth over time. This is not only due to the increased cooling of the blowing agent-loaded polymer melt after the die outlet in the extrusion process, but also mainly due to the changing elongational deformation behavior at the bubble wall over time. In [36], the authors were able to show that the time range of viscosity-driven bubble growth is highly transient. The Hencky strain increases strongly with the expansion of the blowing agent, and thus with increasing bubble growth over time, whereas the strain rate drops rapidly from very high values to almost zero. Exactly this behavior is shown in Figure 10 for all predictions, with an increasing Hencky strain and decreasing strain rate, and thus increasing transient equibiaxial elongational viscosity at the bubble wall over time. Additionally, the typical dependence of temperature on viscosity is illustrated in Figure 10, and thus by the model. This means that the prevailing transient equibiaxial elongational viscosity must be lower at the bubble wall at an increased temperature at a constant blowing agent concentration. This can be seen for the predictions for PS in Figure 10a as well as for PLA in Figure 10b.

### 4.4. Prediction of the Viscosity-Driven and Diffusion-Controlled Bubble Growth Behavior over Time

**Figure 11:**
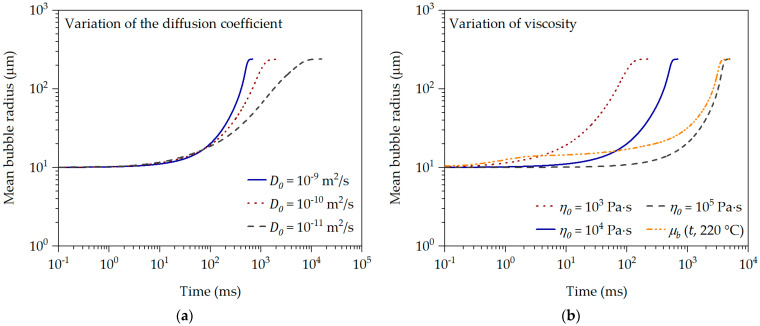
Bubble growth simulation in dependency of diffusion coefficient (**a**) and viscosity (**b**).

The single-cell model described in Section 1 is used to analyze the influence of viscosity and diffusion coefficient on the initial (directly after nucleation of the bubble), and thus viscosity-driven, bubble growth behavior. For this purpose, the following input variables of the bubble growth model were selected exemplarily are kept constant for PS at 220 °C: molecular weight of the blowing agent $M_{N_{2}}$ = 0.02801 kg/mol, ambient pressure $p_{a}$ = 10^5^ Pa, Henry’s solubility constant $H_{k}$ = 1·10^−9^ Pa^−1^, initial bubble radius $R_{0}$ = 10 μm, initial influence cell radius $S_{0}$ = 100 μm, initial gas pressure $p_{G,0}$ = 1·10^6^ Pa, initial N_2_ concentration $c_{0}$ = 0.23 wt.-% N_2_ and surface tension $\sigma_{O}$ = 0.03 N/m. The viscosity and the diffusion coefficient are varied as shown in Figure 11 in order to evaluate the influence on the initial bubble growth behavior. In addition, the single-cell model was compared with and confirmed by the literature data, such as that of Tuladhar and Mackley [19].

The variation of the diffusion coefficient (see Figure 11a) clearly shows that a higher diffusion coefficient accelerates bubble growth. This behavior is also shown by Hu et al. [65]. Interestingly, an increase in the diffusion coefficient hardly influences the onset (approximately 10 ms) of bubble growth. This clearly shows that the diffusion-controlled bubble growth behavior only comes into effect from a later point in time of bubble growth. This is also described by Taki [66], for example.

**Figure 12:**
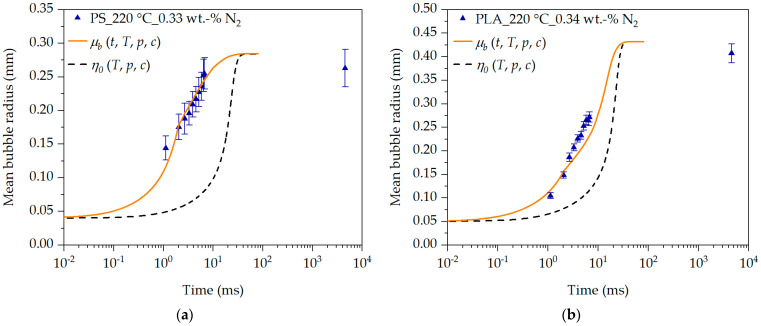
Bubble growth simulation in dependency of the transient equibiaxial elongational viscosity function at the bubble wall during bubble growth at exemplary process conditions for PS (**a**) and PLA (**b**).

The analysis of the influence of viscosity (see Figure 11b) on the bubble growth behavior shows that the onset of bubble growth and its speed are significantly inhibited at higher viscosity compared to a lower viscosity. This behavior is also shown by Yao et al. [32]. However, if the transient equibiaxial elongational viscosity function for PS at 220 °C and 0.23 wt.-% N_2_ is used for the bubble growth simulation instead of a constant or representative viscosity, it becomes clear that the initial bubble growth time range ≤ 1·10^1^ ms is clearly viscosity-driven and shows a strong dependence on the transient equibiaxial elongational viscosity. The transient equibiaxial elongational viscosity function at the bubble wall shows values between approx. 985 Pa·s and 6.2·10^5^ Pa·s in the time range between 1.1 ms and 800 ms in Figure 10a. Thus, knowledge of the transient equibiaxial elongational viscosity function prevailing at the bubble wall is essential to describe the initial, and thus viscosity-driven, bubble growth behavior over time. This becomes particularly evident when the bubble growth predictions are compared with the experimentally determined initial and mean bubble growth behavior over time using the data in [36] (previously published by the authors) and the description in Section 2.3. The initial values and physical input variables used for the bubble growth predictions in Figure 12 are found in Table 1.

The results clearly show that the transient equibiaxial elongational viscosity function at the bubble wall (see Figure 10) allows an exact description of the experimentally determined initial and mean bubble growth behavior in Figure 12 over time. If the zero-shear viscosity, and thus a representative viscosity (shifted to the corresponding process conditions regarding T, p and c), is used for this purpose instead, no description of the bubble growth behavior is given with otherwise identical initial bubble growth model input values for PS and PLA. This clearly demonstrates that the initial bubble growth behavior is strongly viscosity-driven and that knowledge of the transient equibiaxial elongational viscosity function $\mu_{b}\left(t,\dot{\varepsilon },T,p,c\right)$ prevailing at the bubble wall is essential for bubble growth analysis and prediction. Based on the model presented in this work and the determination of the deformation behavior at the bubble wall in [36] (previously published by the authors), the transient equibiaxial elongational viscosity function at the bubble wall of different polymers during bubble growth can be described precisely and realistically.

## 5. Conclusions

In this work, a rheological model was presented to describe the transient equibiaxial elongational viscosity function at the bubble wall during the bubble growth process. For this purpose, a transient uniaxial calibration of the so-called MSF model is required, whereby the transient equibiaxial elongational viscosity function can then be described by changing the mode of deformation parameter in the model. The influence of temperature, pressure and blowing agent concentration on the transient elongational viscosity during bubble growth is described by superposition principles.

The model-based description and prediction of the transient uniaxial elongational viscosity is holistically possible by using the LMSF (for linear polymers) and the MSF (for long-chain branched polymers) models by defining the non-linear LMSF and MSF model parameters. The results showed that with a single choice of the non-linear LMSF and MSF model parameters, an accurate prediction of the entire transient uniaxial elongational viscosity measurement is possible for all investigated Hencky strains, strain rates and temperatures. Furthermore, the MSF model allows the prediction of the onset and progression of strain-hardening as a function of temperature, strain rate and Hencky strain.

The holistic and generally valid description of the rheological behavior under an elongational deformation of blowing agent-loaded polymer melts assumes that the superposition principles determined under a shear deformation can be transferred to an elongational deformation. Thus, by coupling the superposition principles with the LMSF and MSF models, it was shown that a master curve of the transient uniaxial elongational viscosity measurement data can be created under freely chosen conditions regarding the temperature, pressure and blowing agent concentration. The reached transient uniaxial and equibiaxial master curve was successfully described with the extended LMSF and MSF models for PS and PLA. Therefore, a model-based holistic and generally valid description of the transient equibiaxial elongational viscosity function prevailing at the bubble wall during bubble growth over time was achieved for the first time. Furthermore, by using the transient equibiaxial elongational viscosity function at the bubble wall during bubble growth, it was possible to describe the initial, and thus the viscosity-driven and diffusion-controlled, bubble growth behavior over time directly after nucleation using the single-cell model.

In future, the model should be validated with other polymers to further prove its general validity. Instead of the single-cell model, further bubble growth models could be used in combination with the nucleation theory, so that the nucleation and bubble growth behavior could be described holistically with the developed transient elongational viscosity model. This would allow the simultaneous analysis of all nucleated bubbles in the blowing agent-loaded polymer melt, making it possible to predict the overall blowing agent expansion over time, and thus the overall bubble growth within a foamed part.

## Figures and Tables

**Table 1:** Initial input values for the application of the bubble growth model for PS and PLA.

	PS	PLA
Temperature in °C	220	220
$N_{2}\;\mathrm{concentration}\;c_{0}$ in wt.-% N_2_	0.33	0.34
$\mathrm{Initial}\;\mathrm{bubble}\;\mathrm{radius}R_{0}$ in mm	0.05	0.04
$\mathrm{Initial}\;\mathrm{influence}\;\mathrm{cell}\;\mathrm{radius}\;S_{0}$ in mm	0.078	0.116
$\mathrm{Gas}\;\mathrm{pressure}\;p_{G,0}$ in bar	110	146
$\mathrm{Viscosity}\;\eta_{0}\left(T,p,c\right)$ in Pa·s	12.558	9.395
Melt density ρ kg/m^3^	926.7	1067
$\mathrm{Diffusion}\;\mathrm{coefficient}\;D_{0}$ in m^2^/s [54,55,56]	2.36·10^−9^	4.60·10^−9^
$\mathrm{Surface}\;\mathrm{tension}\;\sigma_{O}$ in N/m [57,58]	0.0289	0.0251
$\mathrm{Henry}’s\;\mathrm{solubility}\;\mathrm{constant}\;H_{k}$ in Pa^−1^ [54,55,59]	2.29·10^−10^	3.22·10^−10^

**Table 2:** DE, LMSF and MSF model parameters.

	DE		LMSF		MSF	
	β	$f_{max}^{2}$	β	$f_{max}^{2}$	β	$f_{max}^{2}$
PS	1	1	1	20	-	-
PLA	1	1	1	30	1.6	30

## Data Availability

Data are contained within the article and in the supplementary publications [11,36,37] of the authors.
